# Risk groups defined by Recursive Partitioning Analysis of patients with colorectal adenocarcinoma treated with colorectal resection

**DOI:** 10.1186/1471-2288-12-2

**Published:** 2012-01-03

**Authors:** Yun-Jau Chang, Li-Ju Chen, Yao-Jen Chang, Kuo-Piao Chung, Mei-Shu Lai

**Affiliations:** 1 Graduate Institute of Health Policy and Management, College of Public Health, National Taiwan University, Taipei, Taiwan; 2 Department of General Surgery, Zhong-Xing Branch, Taipei City Hospital, Taipei, Taiwan; 3 Department of General Surgery, National Taiwan University Hospital, Taipei, Taiwan; 4 Department of Ophthalmology, HepingFuyou Branch, Taipei City Hospital, Taipei, Taiwan; 5 Department of Surgery, Taipei Branch, Buddhist Tzu Chi General Hospital, Taipei, Taiwan; 6 Graduate Institute of Epidemiology and Preventive Medicine, College of Public Health, National Taiwan University, Taipei, Taiwan

**Keywords:** Recursive Partitioning Analysis, Colorectal Cancer, Survival Analysis

## Abstract

**Background:**

To define different prognostic groups of surgical colorectal adenocarcinoma patients derived from recursive partitioning analysis (RPA).

**Methods:**

Ten thousand four hundred ninety four patients with colorectal adenocarcinoma underwent colorectal resection from Taiwan Cancer Database during 2003 to 2005 were included in this study. Exclusion criteria included those patients with stage IV disease or without number information of lymph nodes. For the definition of risk groups, the method of classification and regression tree was performed. Main primary outcome was 5-year cancer-specific survival.

**Results:**

We identified six prognostic factors for cancer-specific survival, resulting in seven terminal nodes. Four risk groups were defined as following: Group 1 (mild risk, 1,698 patients), Group 2 (moderate risk, 3,129 patients), Group 3 (high risk, 4,605 patients) and Group 4 (very high risk, 1,062 patients). The 5-year cancer-specific survival for Group 1, 2, 3, and 4 was 86.6%, 62.7%, 55.9%, and 36.6%, respectively (p < 0.001). Hazard ratio of death was 2.13, 5.52 and 10.56 (95% confidence interval 1.74-2.60, 4.58-6.66 and 8.66-12.9, respectively) times for Group 2, 3, and 4 as compared to Group 1. The predictive capability of these grouping was also similar in terms of overall and progression-free survival.

**Conclusion:**

The use of RPA offered an alternative grouping method that could predict the survival of patients who underwent surgery for colorectal adenocarcinoma.

## Background

Adenocarinoma is the most commonly seen malignancy of colon and rectum, which ranks the third leading cause for cancer death both in USA and Taiwan as well as the fourth cause worldwide [1]. Although the diagnostic instrument and treatment modality had made a huge progress leap in recent decade, the survival outcome of colorectal cancer patients didn't keep up the identical or similar pace by multiple factors [2]. Clinical practice guideline and performance measurement came up with the impetus to formalize clinicians' daily practice and possibly improve patients' survival thereafter. The most frequently accepted prognostic factor is TNM staging system, but for real world, there are many factors beyond TNM staging that can confound the patients' survival. Hence a risk group study may hopefully yield substantial information that is succinct and easily understood by researchers, providers, practitioners, patients, and even the policy makers to make appropriate choices.

Recursive partitioning analysis (RPA), a method of classification, was initially described by the Radiation Therapy Oncology Group and was intended to provide a way that divide patients into homogenous groups based on the length of survival [3]. Advantages of this method include not only making fewer modeling assumptions, but also establishing procedure that adapts to missing values through the use of surrogate measures [4]. Currently, sophisticated computer system offers a good solution possible for these tedious computations.

In this study, the main objective was to assign different prognostic groups with regard to cancer-specific survival derived from RPA among patients with newly diagnosed colorectal adenocarcinoma who underwent colorectal resection for cancer surgery from a population-based data. We also compared these groups regarding three different types of survival (overall survival, progression-free survival and cancer-specific survival)

## Methods

### Study population

This study consisted of a consecutive series of 15,731 patients who were newly diagnosed with colorectal cancer during the period of January 2003 to December 2005 from a population-based database, Taiwan Cancer Database, and linkage with TCDB to 2003-2009 Death Registries. Taiwan Cancer Database (TCDB) was a nationwide program that accounted for about 60% of patients with six cancer types (breast, colon, liver, lung, cervical and buccal cancer) per year and served as a good source for academic research [1]. We identified colorectal cancer patients newly registered into the TCDB according to ICD-O-3 (International Classification of Diseases for Oncology, third revision) code C18.0 (cecum)-C21.8 (rectum). Exclusion criteria included patients with stage IV, cancer at anus, pathologic report beyond adenocarcinoma, without colorectal resection, and unknown specification of cancer stage as well as unavailability of lymph node information. We also excluded patients whose survival status cannot be verified as of December 31, 2009. This study was approved by the institutional review board (IRB) at College of Public Health, National Taiwan University.

### Study outcomes

The primary end point was 5-year survival including overall survival, progression-free survival and disease-specific survival. Overall survival rate denoted the percentage of patients who were still alive for a certain period of time after surgery for colorectal cancer. Progression-free survival rate denoted the percentage of patients who were still without any signs of colorectal cancer for a certain period of time after surgery for colorectal cancer. Cancer-specific survival rate (or disease-specific survival) referred to the percentage of patients who had not died from colorectal cancer or metastasis for a certain period of time after surgery for colorectal cancer. All survival rates were calculated from the day of surgery (colorectal resection).

### Statistical analysis

We used classification and regression (CRT) method, developed for binominal data, in the analysis of RPA [5]. This technique is a nonparametric methodology that creates a decision tree with respect to prognostic factors and their interactions which are most important in determining the outcome. A parent node would split into child nodes that are as homogenous as possible to dependent variables. The split also followed the rules that the corresponding cut-off points with the minimal P value, provided the minimal P value was < = 0.0001 and that the number of patients within the child node was at least 50. Then a tree-based model composed of nodes was fashioned by recursively partitioning the study cohort. During the RPA process of this study, a set of variables had been evaluated as prognostic factors, including: age (split at 50, 60, 70 years), gender (male vs. female), comorbidity (Deyo's modified version of the Charlson comorbidity index - CCI) [6], tumor location (colon, sigmoid, rectum), number of lymph nodes retrieved and examined (continuous), distance of surgical margin to tumor (positive or < 0.2 cm, 0.2 cm to 1 cm, > 1 cm), depth of tumor invasion (submucosa, muscularis propria, into subserosa, invasion to other organs), pathological tumor size (0-2 cm, 2-3 cm, 3-4 cm, 4-6 cm, > 6 cm), histopathology (adenocarcinoma vs. mucinous adenocarcinoma or signet ring cell adenocarcinoma), TNM stages (stage I, stage II, stage III, based on the classification of sixth edition of American Joint Commission on Cancer Staging Manual), fluorouracil-based chemotherapy (yes vs. no), radiotherapy (yes vs. no). Because CRT did not stop in the middle of the tree-growing process, we pruned tree to avoid overfitting by setting maximum difference in risk as 1. Also for validation, we randomly assigned 50% cohort as training sample (for model building) and other 50% as test sample (for model validation).

After CRT algorithm, several terminal nodes were created and these nodes would be combined into a group when the significance level of comparison between two terminal nodes was > 0.05. With these RPA group, we used paired t-test to determine whether the 5-year overall, 5-year progression-free and 5-year disease-specific survival differed significantly between these RPA groups. The Kaplan-Meier method was used to calculate cumulative survival. Differences in cumulative survival between two groups were tested by the log-rank test. All P values were two sided and a P < 0.05 was considered statistically significant. We used PASW Statistics 18 as statistic software for all of the analyses reported in this study.

## Results

**Figure 1:**
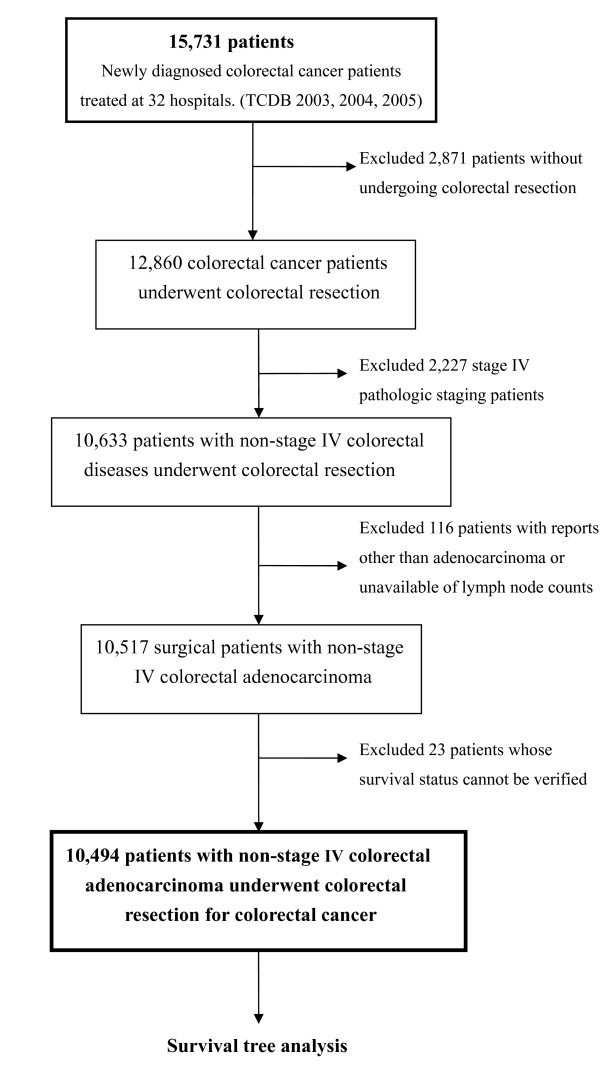
**Schema of patients'enrollment in this study**. (TCDB: Taiwan Cancer Database).

Of 15,731 newly diagnosed colorectal cancer patients, there were 12,860 patients who had undergone colorectal resection for colorectal cancer (Figure 1). Among them, 2,126 patients (16.7%), 4,008 patients (31.5%), 4,383 patients (34.4%) and 2,227 patients (17.5%) presented with stage I, stage II, stage III and stage IV disease, respectively. We excluded 2,227 patients with stage IV disease, 116 patients with pathological report other than adenocarcinoma or unavailable of lymph node counts, and 23 patients whose survival status could not be verified. The resulting cohort, 10,494 patients, who underwent colorectal resection in thirty-two major hospitals or cancer centers were eligible to enter into this survival tree analysis. The majority of patients were older than 70 years of age (39.8%, 4,173/10,494) and comorbidity indexes were > = 3 (52.5%, 5,509/10,494) (Table 1). Among these patients, male patients were slightly more than female patients (56.6%, 5,937/10,494). Rectum was found to be the more frequent site of tumor location. About 63.3% (6,644/10,494) patients had no less than twelve lymph nodes examined and 59.0% (5,816/9,855) of pathology reports proved that surgical margin was free of tumor for more than 1 cm microscopically. In terms of involvement depth and tumor size, most patients (60.2%, 6,315/10,494) had cancer penetrated through the muscularis propria of colorectal wall into the subserosa and tumors grew larger than 4 cm (61.7%, 6,479/10,494). Variants of adenocarcinoma (such as mucinous adenocarcinoma or signet ring cell adenocarcinoma, etc.) occurred in only about 4.6% (478/10,494) of resected specimens. Stage III comprised of 41.7% (4,378/10,494) patients, followed by stage II which comprised of 38.1% (4,002/10,494) patients. Nearly half patients received chemotherapy 48.7% (5,109/10,494). However, much less patient underwent radiotherapy [10.7% (1,127/10,494)]. The duration of follow-up time was 0- 84 months with a mean of 48.9 months. P value of log-rank test for all factors was less than 0.01 (except number of lymph node examined, P = 0.013) when 5-year cancer-specific survival was designated as dependent variable. Paradoxically, the hazard ratio of radiotherapy vs. no radiotherapy was 1.21.

**Table 1:** Demographic and treatment of colorectal adenocarcinoma patients

	N	Cumulative survival (%) not died of cancer at 5-years	HR	95.0% CI	Significance*
Gender					0.000
male	5,937	69%	1.00		
female	4,557	73%	0.86	0.80-0.93	
AGE					0.000
> 50 yrs, < = 60 yrs	1,994	76%	1.00		
< 50 yrs	1,516	75%	1.19	1.03-1.38	
> 60 yrs, < = 70 yrs	2,811	73%	1.14	1.01-1.30	
> 70 yrs	4,173	64%	1.64	1.46-1.84	
Co-morbidity (CCI)					0.000
CCI = 0+1	952	87%	1.00		
CCI = 2	4,033	78%	1.51	1.24-1.85	
CCI > = 3	5,509	62%	1.92	1.58-2.33	
Tumor location					0.000
rectum	3,967	70%	1.00		
sigmoid colon	3,115	72%	0.84	0.77-0.93	
colon (except sigmoid colon)	3,230	70%	1.02	0.93-1.13	
overlapping or unspecified	182	61%	1.17	0.90-1.53	
Number of lymph node examined					0.013
< 12	3,850	70%	1.00		
> = 12	6,644	72%	0.91	0.84-0.98	
Surgical margin					0.000
< = 2 mm	657	57%	1.00		
> 2 mm, < = 1 cm	3,382	69%	0.57	0.49-0.66	
> 1 cm	5,816	75%	0.47	0.41-0.54	
Depth of tumor invasion					0.000
submucosa	958	89%	1.00		
muscularis propria	1,613	82%	1.26	0.73-2.18	
through the muscularis propria into the subserosa	6,315	68%	1.77	1.06-2.95	
directly invades other organs or structures	1,608	58%	2.76	1.62-4.70	
Tumor size					0.001
0-2 cm	625	79%	1.00		
> 2.0, < = 3 cm	1,323	75%	0.82	0.66-1.02	
> 3.0, < = 4 cm	2,067	72%	0.81	0.66-1.00	
> 4.0, < = 6 cm	3,889	69%	0.82	0.67-1.01	
> 6.0 cm	2,590	66%	0.98	0.80-1.21	
Pathology					
adenocarcinoma	10,016	71.6%	1.00		0.000
mucinous or signet ring cell adenocarcinoma	478	62.9%	1.50	1.27-1.76	
TNM stage					0.000
stage I	2,114	88%	1.00		
stage II	4,002	76%	1.23	0.96-1.58	
stage III	4,378	56%	3.05	2.39-3.89	
Radiotherapy					0.004
no	9,367	71%	1.00		
yes	1,127	67%	1.21	1.07-1.38	
Chemotherapy					0.000
no	5,385	68%	1.00		
yes	5,109	73%	0.67	0.62-0.73	

N: number; HR: hazard ratio; CI: confidence interval; CCI: Charlson comorbidity index; classification of TNM stage based on AJCC edition 6; *: log-rank P value

**Figure 2:**
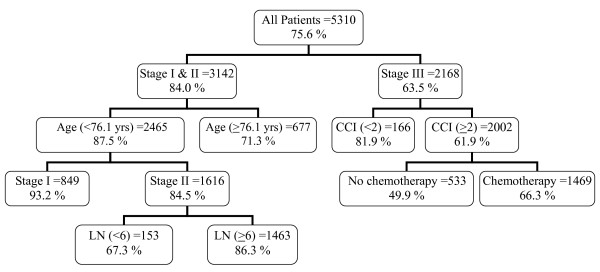
**Decision tree constructed by recursive partitioning analysis (training sample)**.

We started the RPA with training sample of 5310 patients with 1,296 patients (24.4%) died in the study period. Tumor staging (TNM system) was the most important factor that yielded a segment of 3,142 patients (16.0% dead) with stage I & II disease and a segment of 2,168 patients (36.5% dead) with stage III disease (p < 0.001). The same procedure continued following this splitting algorithm (Figure 2). In the left segment, patient's age appeared to be the strongest factor (p < 0.001), which yielded a subgroup of 2,465 patients with age < 76.1 years (12.5% dead) and a subgroup of 677 patients with age ≥ 76.1 years (28.7% dead) (p = 0.001). No further split was possible in the node of stage I & II disease and age ≥ 76.1 years due to minimal criteria. The node with stage I & II disease and age < 76.1 years could be split into a subgroup of 849 patients with stage I disease (6.8% dead) and a subgroup of 1,616 patients stage II disease (15.5% dead) (p < 0.001). No further split was possible in the node of stage I disease and age < 76.1 years due to minimal criteria. But we could split the node with stage II disease and age < 76.1 years into a subgroup of 153 patients who had number of lymph nodes examined < 6 (32.7% dead) and a subgroup of 1,463 patients who had number of lymph nodes examined ≥ 6 (13.7% dead), both of which were terminal nodes.

**Figure 3:**
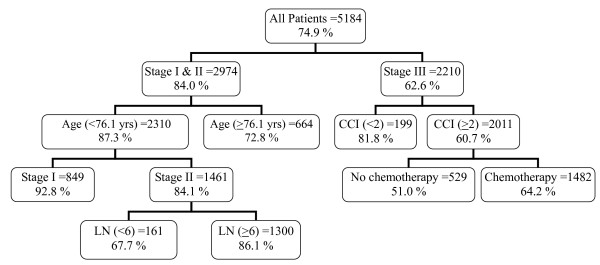
**Decision tree constructed by recursive partitioning analysis (test sample)**. The plots of recursive partitioning analysis (Figure 2 and Figure 3) were obtained from 10,494 patients who were documented to have adenocarcinoma from colon and rectum (anus was not included) and underwent colorectal resection for cancer. (CCI: Charlson comorbidity index; LN: number of lymph nodes; yrs: years; the number in the upper half of box indicated number of patients; the percentage in the upper half of box indicated cancer-specific survival percentage).

In the right segment, comorbidity score (CCI) seemed to be the strongest factor, by which yielded a subgroup of 166 patients with CCI < 2 (18.1% dead) and a subgroup of 2002 patients with CCI ≥ 2 (38.1% alive) (p = 0.001). The node that patients with stage III and CCI < 2 was a terminal node since no further split was possible. The node that patient with stage III and CCI ≥ 2 could be further split into a subgroup of 533 patients who didn't have chemotherapy treatment (50.1% dead) and a subgroup of 1,469 patients who ever have been treated with chemotherapy treatment (33.7% dead), both of which were terminal nodes. The results of the RPA process were validated with a test sample of 5,184 patients with colorectal cancer which were independent of the model building training sample (Figure 3). Both results were closely correlated.

Thus five prognostic factors were identified (namely, TNM staging, age, comorbidity, number of lymph nodes examined, chemotherapy) for cancer-specific survival, resulting in seven terminal nodes. Based on mean survival time of the terminal nodes, we were able to categorized four risk groups (Table 2). Group 1 (mild risk) consisted of 1,698 patients who had stage I colorectal cancer and age < 76.1 years (119 deaths in the study period). Group 2 (moderate risk) consisted of 3,129 patients who had stage II colorectal cancer, age < 76.1 years and number of lymph nodes examined ≥ 6, or stage III colorectal cancer with CCI < 2 (449 deaths in the study period). Group 3 (high risk) consisted of 4,605 patients who had stage I&II colorectal cancer and age ≥ 76.1 years or stage II colorectal cancer, age < 76.1 years and number of lymph nodes examination < 6, or stage III colorectal cancer CCI ≥ 2 with chemotherapy (1,502 deaths in the study period). Group 4 (very high risk) consisted of 1,062 patients who had stage III colorectal cancer, CCI ≥ 2 and without chemotherapy (525 deaths in the study period).

**Table 2:** Assignment of Recursive Partitioning Analysis (RPA) Groups

RPA class	Definition (s)	Patients	Events
Group 1 (mild risk)	stage I colorectal cancer and age < 76.1 years,	1,698	119
Group 2 (moderate risk)	stage II colorectal cancer, age < 76.1 years and LN examined > = 6 stage III colorectal cancer, CCI < 2	3,129	449
Group 3 (high risk)	stage I & II colorectal cancer, age ≥ 76.1 years stage II colorectal cancer, age < 76.1 years and LN examined < 6 stage III colorectal cancer, CCI > = 2, with chemotherapy	4,605	1,502
Group 4 (very high risk)	stage III colorectal cancer, CCI > = 2, without chemotherapy	1,062	525

RPA: recursive partitioning analysis; CCI: Charlson comorbidity index; LN: lymph node

**Figure 4:**
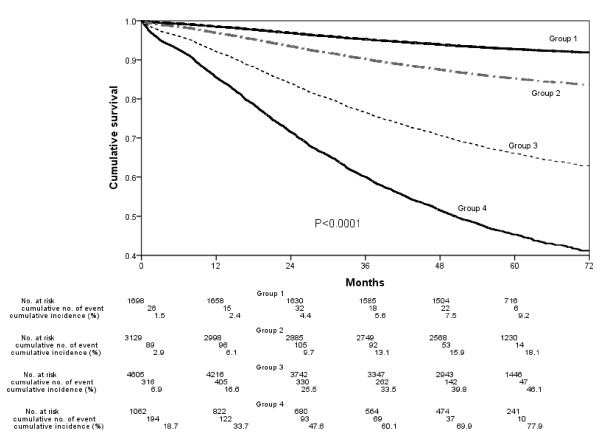
**Survival analysis (cancer-specific survival as outcome) with Kaplan-Meier plot shows significant difference between groups (p < 0.0001)**. (No.: number).

Cancer-specific survival analysis using Kaplan-Meier plot and log-rank test revealed significant differences among groups (p < 0.0001, Figure 4). In addition, we also utilized this RPA grouping classification to test the effects on predicting overall and progression-free survivals (3-year and 5-year respectively). Results showed good discriminating capability for this grouping classification to easily predict each outcome for all endpoints (Table 3).

**Table 3:** The RPA groups and survival

	RPA Group				
	Group 1	Group 2	Group 3	Group 4	P value
**Overall survival**					
3-yrs (%)	91.5	84.1	65.7	45.2	< 0.001
5-yrs (%)	85.6	78.3	54.8	35.1	< 0.001
HR	1.00	1.70	4.12	7.88	
95% CI		1.44-1.99	3.56-4.78	6.71-9.25	
**Progression-free survival**					
3-yrs (%)	94.2	86.0	69.4	51.7	< 0.001
5-yrs (%)	90.4	81.4	60.6	41.7	< 0.001
HR	1.00	2.25	5.42	9.91	
95% CI		1.85-2.74	4.52-6.51	8.16-12.11	
**Cancer-specific survival**					
3-yrs (%)	94.5	87.5	70.5	52.1	< 0.001
5-yrs (%)	91.1	83.2	61.9	43.2	< 0.001
HR	1.00	2.13	5.52	10.56	
95% CI		1.74-2.60	4.58-6.66	8.66-12.90	

RPA: recursive partitioning analysis; yrs: years; HR: hazard ratio; CI: confidence interval

## Discussion

This study explored the risk group definition of patients with colorectal adenocarcinoma after surgery by CRT algorithm. Risk group definition by RPA algorithm has been already proposed to predict outcomes of several benign and malignant diseases [3-5], but it was still rare for colorectal cancer as well as other gastrointestinal malignancy. For patients with rectal adenocarcinoma, Zolbec et al. ever used this technique to identify a predictive model with regard to patients' responses to preoperative radiotherapy from several molecular factors [7]. An empirically useful model that predict survival outcome credibly allows important clinical implication for patients, surgeons and stakeholders to make appropriate treatment decisions. In this study, the results yielded five prognostic factors, which could be further used to define four risk groups. These four RPA groups not only differed significantly with regard to cancer-specific survival but also provided prognostic significance concerning with progression-free survival as well as overall survival.

Traditionally, a prognostic or risk factor can be easily identified through univariate or multivariate analyses from Cox proportional hazards model as Table 1 demonstrated in this study. It may be possible to predict a survival probability of a certain prognostic factor through Cox proportional hazards model, however, it is always difficult to interpret or predict a patient's cumulative risk for a given set of prognostic factors. In actual daily practice, patients usually present with a lot of prognostic or risk factors, especially when there are interaction terms involved. Hence, provision a useful and informative risk group definition for empirical use is a tough task. Furthermore, the cut-off values of defining risk groups in the hazard model often are arbitrary [5]. Theoretically, multivariate regression model offers hazard ratio for entire population while RPA allows different prognostic factors for different branch of the tree model. Therefore, RPA is a better statistic methodology when there are interactions between prognostic (or risk) factors.

Several studies have documented the association between patient attribute, tumor characteristics, process (treatment), pathological finding and the survival of colorectal cancer. Prognostic or risk factors frequently observed for survival are gender [8,9], age [9], comorbidity [10], number of lymph nodes examined [11-13], tumor size [14], tumor TNM staging [12,15], depth of tumor invasion [16], safety surgical margin [17], chemotherapy [18-21] and radiotherapy [22]. Except radiotherapy, the prognostic significance of these variables was confirmed in our study by Cox proportional hazard model. However, the impact of predictability of radiotherapy with regard to cancer-specific survival in the hazard model, estimated through all colorectal cohort (not stage III rectal cancer only), may decreased even in the opposite direction as described in the Method section.

Tumor stage was still the most important prognosticator throughout this tree-structured model for cancer-specific survival of patients with colorectal cancer. Patients with stage III disease survived much shorter than patient with stage I or II disease. For those patients with negative lymph node status (stage I & II disease) in the left segment of Figure 2, we identified that age as well as number of lymph nodes examined were associated with patients' long-term survival. For those patients with positive lymph node status (stage III disease only) in the right segment, we identified comorbidity index and chemotherapy were associated with patients' long-term survival. In other words, age was a prognostic factor for patient with stage I disease; while age and harvest lymph nodes ≥ 6 might predict long-term survival for patient with stage II disease. Comorbidity index and chemotherapy were the most important prognostic factors for patient with stage III disease. We noted in the subset of patients with stage III disease and CCI ≥ 2, chemotherapy had been identified as one of the most important prognostic factors postoperatively for survival after colorectal surgery. The benefit was about 15% improvement in survival. Compared to prior report, Mamounas et al. had found chemotherapy resulted in an increase of 18% survival in overall survival for stage III colon cancers [23]. In a meta-analysis investigating the usage of chemotherapy, Benson et al. had discovered the 14% decrease in 5-year mortality rate for stage II colon cancer patients who received 5-FU based chemotherapy [24]. However, our data failed to prove this advantage for patient with stage II disease.

No matter where the tumor located (colon or rectum), comorbidity exhibited an important predictive value for long-term survival. Our results echoed the study of Klabunde et al., who had demonstrated that comorbidity status can predict long-term survival of patients with four types of malignancy including colorectal cancer [10]. Originally developed by epidemiologists to predict hospital mortality of breast cancer, comorbidity seems to be useful in predicting patient's long-term survival with regard to coexisting medical conditions and malignancies. Our results also elucidated the importance of lymph node harvest of surgery for colorectal cancer, which was recently endorsed or adopted by several academic societies and healthcare management organizations to be a quality metric for colorectal cancer care [25]. Although the number of six lymph nodes deriving by CRT algorithm from our study was different from their minimum requirement of twelve lymph nodes, this might highlight us that harvest enough lymph nodes was an important prognosticator for patients with stage II disease. Several authors suggested that understaging might account for the underling mechanism which explained why inadequate lymph node yield would lead to decreased patients' survival [26,27]. Another unexpected finding in this survival tree model was radiotherapy. Adjuvant therapy (chemotherapy and radiotherapy) had been advocated for certain group of patients with colorectal cancer by several authors as alternatives to improve patient's outcome [28]. While chemotherapy could predict patients' long-term survival in the model, radiotherapy failed to show its importance in this respect. The small volume of patients treated with radiotherapy and the default setting of RPA algorithm might somewhat explain why radiotherapy didn't enter in this model.

There were several forms of survival that could be used to evaluate the consequences of patients underwent certain treatment procedures or interventions, including overall survival, progression-free survival and disease-specific survival [5]. Perhaps one may question why we used cancer-specific survival as the outcome determinant of the model building of RPA in the analysis. Overall survival might be the most frequent used form of survival for its information easily collected. But overall survival focused on patients' survival during a certain period of time regardless of patients' reasons of death being cancer-related condition or not, which probably not truthfully reflect the consequences or outcomes of an intervention given to a specific disease such as cancer. For patients with cancer, especially colorectal cancer (geriatric patients were not uncommon), the cause of mortality might not actually related to colorectal cancer or liver metastasis. Causes of death other than disease-specific cancer death in this study included accident (or suicide), old age, other cancer, diabetes, cardiovascular accident and asthma attack, etc. So we did think the optimum candidate of outcome variable in the model building process in this study was cancer-specific survival.

This study has two contributions toward predicting outcomes after surgical intervention of colorectal cancer. First, we tried to apply classification and regression model for evaluating treatments of colorectal cancer patients and to define risk groups relating to long-term survival. To the best our knowledge, this is the first report regarding tree-structured survival analysis for colorectal cancer surgery using population-based data. Second, we sought to present outcomes in three forms (overall, progression-free and disease-specific survival) simultaneously, which had rarely been shown in related literature.

Several limitations of this method in this study should be mentioned. First, we did not include any molecular marker in RPA process due to unavailability of data, which beyond the range that our population-based data regularly collected. Second, although we try to find out risk group definition at national level, this was not really a 100% nationwide database of all colorectal cancer patients. However, we thought this database (around 60% of all colorectal cancer annually) literally enough in representing the daily situation what we encountered everyday. Third, we're not sure that the findings from our population-based dataset could be extrapolated to other health system where multiple factors might not be the same. After all, the healthcare insurance system in Taiwan is a single-payer system.

## Conclusions

In conclusion, our study demonstrated the utility of classification and regression tree in patients with colorectal adenocarcinoma disease treated with colorectal resection. The risk groups defined by RPA algorithm with regard to cancer-specific survival could also predict 5-year overall and progression-free survival. RPA could be used as an alternative method to study prognosis of cancer. In the future, it may require more studies in other healthcare systems to validate the utility of RPA in colorectal cancer care.

## Competing interests

The authors declare that they have no competing interests.

## Authors' contributions

LJC designed methods, analyzed data, and revised the manuscript. YJC (Yun-Jau Chang) analyzed data, interpreted the results and wrote the manuscript. YJC (Yao-Jen Chang) partook in the interpretation of the results and discussion. KPC and MSL participated in data collection and reviewed the manuscript. All authors read and approved the final manuscript.

## Pre-publication history

The pre-publication history for this paper can be accessed here:

http://www.biomedcentral.com/1471-2288/12/2/prepub
